# Optimization of *Buddleja globosa*-Loaded Polymeric Scaffolds for the Treatment of Biofilm-Infected Wounds

**DOI:** 10.3390/ijms27104240

**Published:** 2026-05-10

**Authors:** Tania F. Bahamondez-Canas, Ivan García-Collao, Pamela Perez-Basaez, Carolina V. Delgado-Gazzoni, Henry E. Garrido-Vera, Ricardo Ceriani, Caroline R. Weinstein-Oppenheimer, Daniel F. Moraga-Espinoza

**Affiliations:** 1 Escuela de Química y Farmacia, Facultad de Farmacia, Universidad de Valparaíso, Gran Bretana 1093, Playa Ancha, Valparaíso 2340000, Chilepamela.perez@estudiantes.uv.cl (P.P.-B.); carolina.delgadog@postgrado.uv.cl (C.V.D.-G.); henry.garrido@uv.cl (H.E.G.-V.); ricardo.ceriani@uv.cl (R.C.); caroline.weinstein@uv.cl (C.R.W.-O.); 2 Centro de Investigación, Desarrollo e Innovación en Productos Bioactivos (CInBIO), Universidad de Valparaíso, Gran Bretana 1093, Playa Ancha, Valparaíso 2340000, Chile; 3 Centro para la Investigación Traslacional en Neurofarmacología (CiTne), Universidad de Valparaíso, Gran Bretana 1093, Playa Ancha, Valparaíso 2340000, Chile

**Keywords:** scaffolds, natural extracts, wound healing, biofilms, infections

## Abstract

Chronic wounds are frequently complicated by biofilm-associated infections that impair healing and limit treatment efficacy. *Buddleja globosa* (BG) exhibits antimicrobial and regenerative properties, making it a promising candidate for advanced wound care. This study aimed to optimize the concentration of a standardized BG extract incorporated into polymeric scaffolds for the treatment of wounds infected with the dual-species biofilm (DSB) of *Pseudomonas aeruginosa* and *Staphylococcus aureus*. Scaffolds containing increasing BG concentrations (BG1 to BG4) were fabricated by lyophilization and characterized in terms of physicochemical properties, antimicrobial activity, and cytocompatibility. Their therapeutic efficacy was further evaluated using an in vitro artificial wound model and a murine model of a DSB-infected wound. BG incorporation significantly influenced the scaffold properties. While BG1–BG3 maintained a comparable structure and mechanical integrity, BG4 exhibited a reduced pore size, swelling capacity, and mechanical resistance. All BG-loaded scaffolds reduced bacterial viability in vitro, with BG4 showing the strongest antimicrobial effect. In vivo, BG2 showed the most consistent overall performance, combining improved wound closure at day 6 with complete re-epithelialization at the endpoint. BG3 improved wound closure at day 6 but did not outperform it in re-epithelialization. In contrast, BG4 did not enhance healing despite its higher antimicrobial activity in vitro. These findings demonstrate that scaffold performance is governed by the interplay between extract loading and physicochemical properties, and that intermediate BG concentrations provide more favorable conditions for tissue repair than higher loadings. This work supports the potential of BG-loaded scaffolds as a therapeutic strategy for biofilm-infected chronic wounds.

## 1. Introduction

Chronic wounds are a challenging healthcare issue requiring advanced care due to the prolonged healing time, high susceptibility to infections, and subsequent economic burden [1]. These wounds primarily affect patients with underlying metabolic, mobility-related, or immune disorders, with venous ulcers, pressure ulcers, and diabetic foot ulcers being the most prevalent types. Multiple factors are known to contribute to wound chronicity, among which the presence of microbial biofilms has been identified in the majority of chronic wounds compared to acute wounds [2,3,4]. Biofilms are structural bacterial communities embedded in a self-produced extracellular polymeric substance that provides protection against antimicrobials and the host immune responses [2,5]. Biofilms exacerbate the inflammatory response and contribute to wound chronicity by arresting the healing process in the inflammatory phase [5]. This evidence has driven the development of wound care strategies focused on biofilm prevention and eradication with promising outcomes [6,7].

Infection control is a critical aspect of wound management, particularly in chronic wounds. Standard care includes frequent wound cleaning, infection control, and the use of appropriate dressings [8]. An ideal wound dressing should provide a barrier from the environment, maintain a moist environment, and allow gas exchange, as oxygen plays a key role in tissue repair [9].

Wound exudate is needed for healing as a physiological fluid that provides nutrients for cell proliferation, but exudate accumulation can be detrimental and cause the maceration of the periulceral area [10]. Advanced wound dressings, such as polymeric scaffolds, have gained attention due to their ability to conform to irregular wound surfaces and provide structural support for cell proliferation while gradually degrading within the wound bed [11]. Moreover, these systems can serve as a platform for controlled drug delivery by releasing actives by diffusion or erosion. However, current therapeutic options remain limited, as antimicrobial agents may exhibit cytotoxic effects on the fragile granulation tissue, hindering the healing process [12,13,14,15].

Natural extracts have emerged as promising alternative source of compounds with antimicrobial and anti-inflammatory properties for wound healing based on their traditional use by different cultures. *Buddleja globosa* (BG) is an example of a medicinal plant traditionally used in Chile and Argentina for the treatment of wounds [16,17]. Its biological activities, such as antioxidant, antimicrobial, and anti-inflammatory effects, make it a potential candidate for its delivery by advanced wound care systems [18,19]. The biological activity of BG has been largely attributed to its high content of polyphenolic compounds, including verbascoside and flavonoids, which exhibit antioxidant, antimicrobial, and anti-inflammatory effects [20,21,22]. These compounds have been reported to modulate reactive oxygen species, inhibit microbial growth, and potentially interfere with biofilm formation and quorum-sensing mechanisms. Such multifunctional activity is particularly relevant in chronic wounds, where persistent inflammation and microbial colonization coexist and mutually reinforce impaired healing.

*Staphylococcus aureus* and *Pseudomonas aeruginosa* are the most prevalent pathogens isolated from chronic wounds. *S. aureus* is more frequently detected, likely due to its predominance in superficial wound layers typically sampled in clinical analysis [23,24]. *P. aeruginosa* is strongly linked to poor healing outcomes due to its ability to form biofilms deeply embedded within the wound bed [25,26,27]. Importantly, coinfection with these pathogens has shown higher virulence and wound healing impairment [28]. DeLeon et al. found that these pathogens synergize during biofilm formation, showing a more robust extracellular substance and enhanced tolerance to antimicrobial treatments when grown together [29,30]. Therefore, models that incorporate polymicrobial biofilms provide a more clinically relevant platform for evaluating therapeutic strategies, as they better reproduce the spatial organization, resistance mechanisms, and host interactions observed in chronic wounds.

In a previous study, we developed polymeric scaffolds composed of varying proportions of gelatin, chitosan, and hyaluronic acid, incorporating a fixed concentration of BG extract. This work identified an optimal polymeric composition that maximized antimicrobial activity against *P. aeruginosa* and *S. aureus,* while maintaining cytocompatibility with human dermal fibroblasts in vitro [31]. Building on these findings, the aim of the present study was to determine the optimal concentration of BG extract within this selected polymeric scaffold by evaluating its physicochemical properties, antimicrobial activity against dual-species biofilms, and therapeutic efficacy in a murine model of infected wounds. This integrated approach allows us to identify formulation parameters that maximize both antimicrobial performance and tissue regeneration.

Despite the promising biological properties of BG extracts, the influence of the extract concentration on the physicochemical properties of polymeric scaffolds and how these changes translate into therapeutic efficacy in biofilm-infected wounds remains poorly understood. In particular, optimizing the balance between antimicrobial activity and scaffold structural integrity represents a key challenge for successful clinical applications.

## 2. Results

### 2.1. Physicochemical Characterization of the Scaffolds

**Figure 1:**
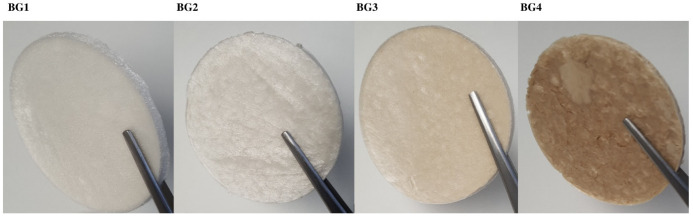
Appearance of BG (Buddleja globosa)-loaded scaffolds obtained by lyophilization. The scaffolds were prepared in 9 cm round Petri dishes and resulted in round scaffolds of 7.5 cm diameter.

Four different BG scaffolds (BG1 to BG4), plus the reference scaffold (S), were obtained by lyophilization. The scaffolds retain an increasing brownish color from the BG extract (Figure 1). These scaffolds had a diameter of about 7.5 cm, a very low density, and increasing average weights related to the BG content (Table 1).

**Figure 2:**
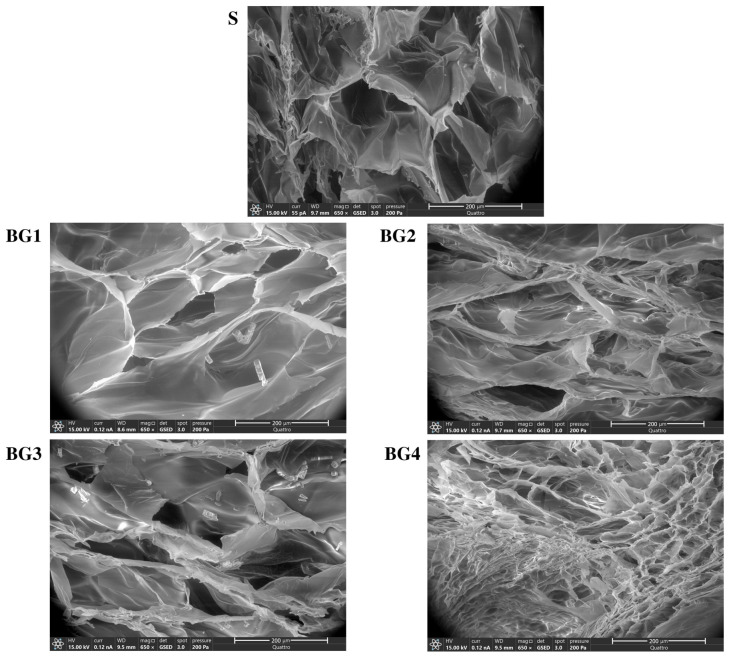
Micrographs of the BG scaffolds. Transversal cuts of representative scaffolds obtained at 650× magnification by FESEM. The scale bar represents 200 µm.

After fabrication, it was observed that the BG extract content significantly influenced the physical properties of the scaffolds. Figure 2 presents cross-sectional images of scaffolds. Scaffold S (without the BG extract) exhibited larger and more rounded pores, whereas scaffolds BG1 to BG3 appeared similar in size but with a slightly flatter pore morphology. In contrast, BG4 was distinguished by a markedly smaller pore size.

**Figure 3:**
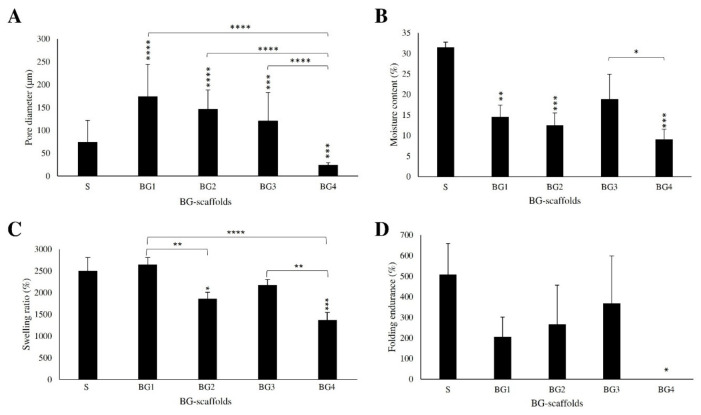
Physical characterization of the BG scaffolds: (**A**) pore diameter, (**B**) moisture content, (**C**) swelling ratio, and (**D**) folding endurance. Symbols above the bars indicate statistical differences with respect to the reference scaffold S (n = 3, mean ± S.D.). * *p* ≤ 0.05, ** *p* < 0.01, *** *p* < 0.001, and **** *p* < 0.0001.

Figure 3 shows the summary of the physicochemical characterization. Scaffold S had the highest moisture content (above 30%) and folding endurance (about 500 folds), and a high swelling ratio (2500%). The average pore diameter (Figure 3A) and moisture content (Figure 3B) decreased significantly when the extract was added. The average pore size and moisture content reached a minimum of 24.1 µm and 9.1% in BG4, respectively. Similarly, it was observed that there was a significant decrease from 2646% (BG1) to 1370% (BG4) in the swelling behavior of the scaffolds with the increase in the extract (Figure 3C). Finally, BG4 scaffold did not withstand any folding (Figure 3D), whereas the folding endurance increased from BG1 to BG3, although the difference was not statistically significant.

Preliminary release experiments performed with BG1 and BG3 demonstrated a measurable release of flavonoid-containing compounds into both PBS and bovine plasma over 6 h (Supplementary Figure S1). BG3 released higher flavonoid levels than BG1, consistent with its higher extract loading. In addition, the release tends to be higher in bovine plasma than in PBS.

### 2.2. In Vitro Antimicrobial Properties and Cytocompatibility

**Figure 4:**
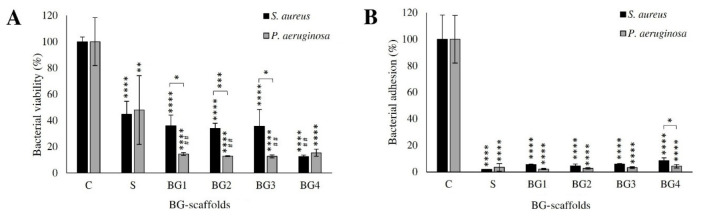
Effect of BG scaffolds on planktonic bacterial viability and adhesion. Scaffolds were incubated with suspension of *P. aeruginosa* and *S. aureus* for 24 h. (**A**) %Bacterial viability within the remaining scaffolds. (**B**) %Bacterial adhesion to the wells (n = 3; mean ± S.D.). Symbols above the bars indicate statistically significant differences with respect to the untreated control (C; *) and the reference scaffold (S; #). * *p* ≤ 0.05, ## *p* < 0.01, ** *p* < 0.01, *** *p* < 0.001, and **** *p* < 0.0001.

After incubating the BG scaffolds with bacterial suspensions, most of the S and some BG1 scaffolds were completely degraded and could not be recovered (Supplementary Figure S2). This qualitative observation suggests that BG extract incorporation may influence scaffold stability under the tested conditions. S significantly reduced the viability of both species by about 50% (*p* < 0.01) (Figure 4A). This reduction in viability was further increased by the addition of the extract in all the scaffolds (BG1 to BG4) (*p* < 0.0001) against *P. aeruginosa* and in BG4 against *S. aureus*. BG4 showed the highest antimicrobial activity on both species (about 15% viability). Finally, all the scaffolds were able to prevent bacterial adhesion to the wells without marked differences among the formulations (Figure 4B).

**Figure 5:**
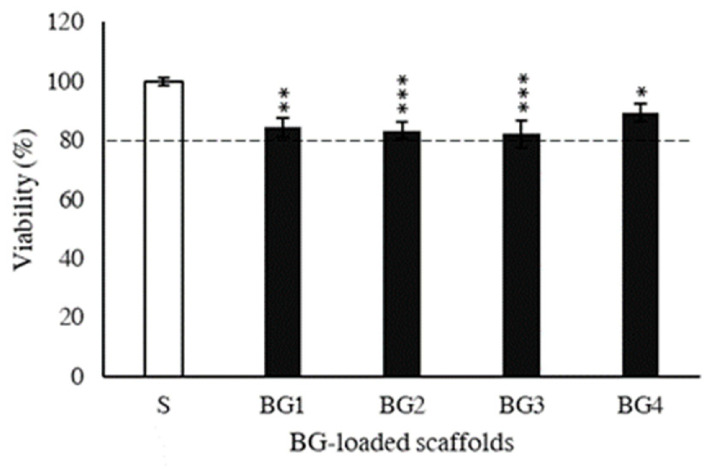
Compatibility of scaffolds with human dermal fibroblasts in vitro. The viability of fibroblasts growing on the scaffolds was determined by a resazurin assay after 72 h of incubation (n = 3; mean ± S.D.). Symbols indicate statistically significant differences with respect to the S group. * *p* ≤ 0.05, ** *p* < 0.01, and *** *p* < 0.001.

The viability of fibroblasts on the reference scaffolds (without the BG extract) represented 100%, and, in all the BG scaffolds, remained above 80% and were comparable among the different formulations without significant differences (Figure 5).

### 2.3. In Vitro Antimicrobial Properties Against Dual-Species Biofilm (DSB)

**Figure 6:**
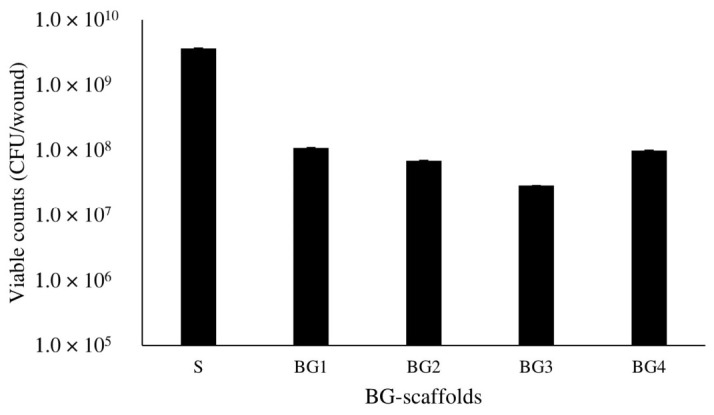
Viability of *P. aeruginosa* and *S. aureus* from dual-species biofilms (DSB) and treated with BG scaffolds in an artificial wound model.

DSB, consisting of *P. aeruginosa* and *S. aureus*, had a gelatinous consistency, yellowish color, and an average mass of 0.5 ± 0.2 g. DSB were fractioned in sterile conditions and sections of 0.15 g were used to inoculate each artificial wound and test the scaffolds. Supplementary Figure S3 shows the artificial wound beds after treatments, where it is observed that none of the treatments were able to prevent bacteria spreading to the surrounding area. The untreated control and S scaffolds show merged bacterial colonies in the characteristic green color which is indicative of *P. aeruginosa* growth. Scaffolds BG1 to BG4 also show this growth but in a more yellow to brownish color that also correlates with the increased BG extract concentration. BG4 shows a marked yellow color towards the agar that could indicate the diffusion of the extract from the scaffold. Figure 6 shows the treatment with the BG scaffolds decreased the number of viable colonies from DSB as compared to the treatment with the scaffolds without the BG extract (S) and this reduction was higher with BG3.

### 2.4. In Vivo Efficacy of the BG Scaffolds on a Murine Model of Infected Chronic Wound

**Figure 7:**
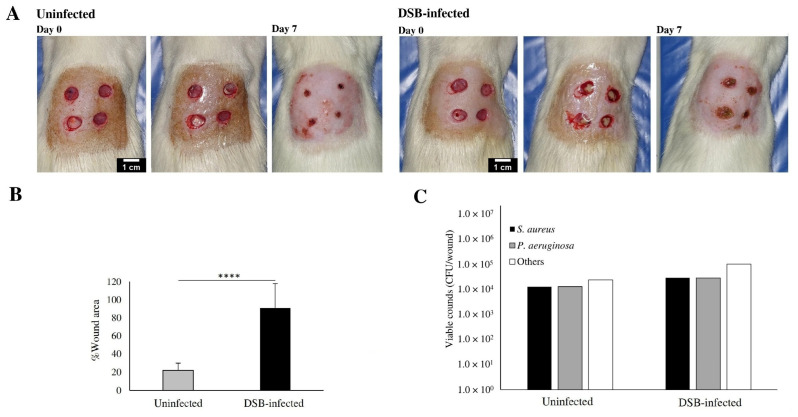
Effect of DSB infection in wound closure: (**A**) pictures of wounds at day 0 (before and after application of the DSB), and after 7 days (scale bar: 1 cm); (**B**) %Wound area of uninfected and DSB-infected wounds after 7 days (**** *p* < 0.0001); and (**C**) CFUs recovered from wound biopsies.

The inoculation of wounds with a preformed DSB is shown to be effective in establishing the infected wound model. The inoculated wounds showed the characteristics signs of inflammation and infection such as edema, reddish, purulent secretions, and periulcer skin maceration. Figure 7 shows the evolution of representative uninfected and infected animals, observing a significant decrease in wound closure. After 7 days, uninfected wounds reached about 80% of closure while DSB-infected wounds only reached about 9% closure (Figure 7A,B), confirming the impact of this model of infection on wound healing.

The recovered viable counts were slightly higher in the DSB-infected wound biopsies compared to the uninfected wounds, but this was not significant as all resulted in the same low order of magnitude (10^4^) compared with the in vitro wound model bacterial load after 24 h of treatment (from 10^7^ to 10^9^) (Figure 7C).

**Figure 8:**
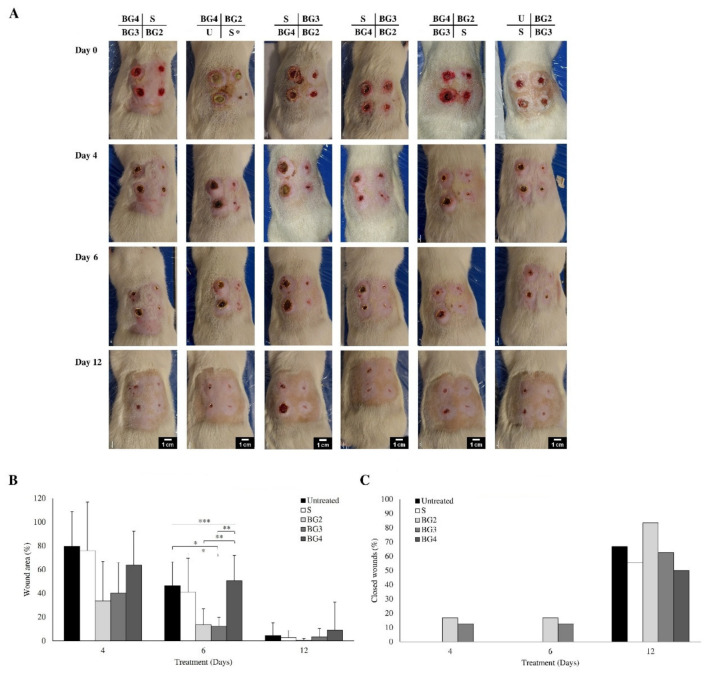
Effects of BG scaffolds on a murine model of biofilm-infected wound. (**A**) Representative progression of infected wounds. Labels on top indicate the treatments randomly assigned to each wound: U (untreated wounds), S (reference scaffold), BG2 to BG4 (BG-loaded scaffolds), and S* (wound removed from the study as it did not showed signs of infection after inoculation) (scale bar: 1 cm). (**B**) %Wound area normalized with respect to the initial area right before application of the treatment. (**C**) %Wounds that achieved full closure (n = 39 wounds). * *p* ≤ 0.05, ** *p* < 0.01, and *** *p* < 0.001.

For the in vivo evaluation of the scaffolds, we selected formulations BG2, BG3, and BG4, along with S, based on their in vitro properties, with BG3 being the scaffold with the overall better performance at this point. The wounds of 11 animals were inoculated with DSB and monitored for 7 days. After this time, the wounds showed signs of infection in different degrees (Figure 8A, Day 0). One animal of the group had to be euthanized before treatment due to the severe progression of the infection and fusion of the wounds. On the other hand, one wound was removed from the study as it did not showed signs of infection and showed significant closure, indicating an unsuccessful induction of the infection (see Figure 8A, Day 0, *). The area of the wounds obtained after cleansing and before the application of the treatment was used to normalize the wound area. The progression of the wound closure is shown in Figure 8A.

On day 4, BG2 and BG3 showed a favorable wound closure trend compared with the S and the untreated groups, although these differences were not statistically significant. By day 6, significant differences were detected, with BG2 showing greater wound closure than the untreated group (*p* < 0.05) and BG3 showing greater wound closure than the S group (*p* < 0.05) (Figure 8B). Finally, after 12 days of treatment, all wounds had comparable areas, irrespective of the treatment, achieving more than 90% of closure overall. When evaluating the number of wounds that achieved complete closure, BG2 stands out having the greatest number of closed wounds at days 4, 6, and 12 (Figure 8C).

**Figure 9:**
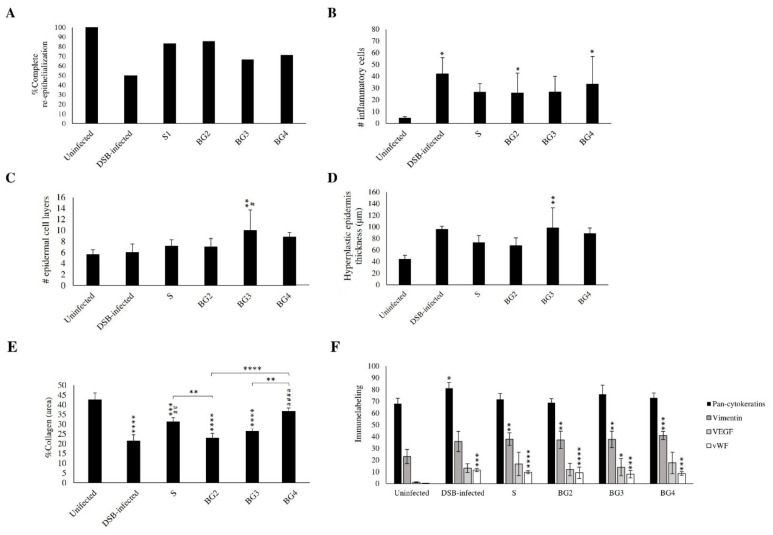
Histological and immunohistochemical assessment of dual-species biofilm (DSB)-infected wound healing. (**A**) %Wounds with complete re-epithelialization. (**B**) Number of inflammatory cells per field. (**C**) Number of epidermal cell layers. (**D**) Hyperplastic epidermic thickness. (**E**) %Collagen deposition. (**F**) Expression of pan-cytokeratin, vimentin, vascular endothelial growth factor (VEGF), and Von Willebrand Factor (vWF). Uninfected and untreated control, DSB-infected, S: Scaffolds without BG extract, BG2, BG3, and BG4 (mean ± S.D.). Symbols indicate significant differences with respect to uninfected group (*) or DSB-infected group (#). *, # *p* < 0.05, **, ## *p* < 0.01, *** *p* < 0.001, and ****, #### *p* < 0.0001.

Finally, Figure 9 shows the results of the histological analysis of wound biopsies at the end of the treatment. BG2 and S were the scaffolds that resulted in the highest number of wounds that achieved complete re-epithelization (Figure 9A). The number of inflammatory cells in the wounded area were comparable among all treatments but lower than untreated wounds (Figure 9B). The number of epidermal cell layers was high in all treated groups but significantly higher in BG3 (Figure 9C), and the height of the hyperplastic epidermis was significantly higher in all groups when compared to the uninfected group, but the BG2 treatment resulted in values compared to the uninfected group (Figure 9D). Collagen deposition analysis showed that DSB infection significantly reduced the collagen-positive area compared with uninfected wounds (Figure 9E). BG4 significantly increased collagen deposition, reaching levels closer to those of uninfected tissue and significantly higher than BG2 and BG3, while no significant differences were observed between S and BG4, suggesting comparable effects.

Immunohistochemical analysis showed a strong pan-cytokeratin expression in both the intact and hyperplastic epidermis, with a higher average positivity in the DSB-infected group (Figure 9F). Vimentin exhibited a strong expression in the cytoplasm of fibroblasts and in the endothelium of blood vessels, with the highest average positivity observed in the BG4 group. VEGF showed a strong expression in the vascular endothelium and a moderate to strong expression in activated fibroblasts, again with the highest levels in the BG4 group. In contrast, the vWF expression was lower than VEGF, with the positive staining limited to a smaller number of blood vessels, and a slightly higher positivity observed in the DSB-infected group. Finally, only the expression of pan-cytokeratins and vWF changed in infected wounds. Pan-cytokeratins were significantly higher and all treatments seemed to normalize it, but this did not occur with vWF, which remains high with all treatments. Vimentin and VEGF significantly increased in the treatments. Representative immunohistochemical images for VEGF, pancytokeratin (AE1/AE3), vimentin, and vWF staining are presented in Supplementary Figure S4.

**Figure 10:**
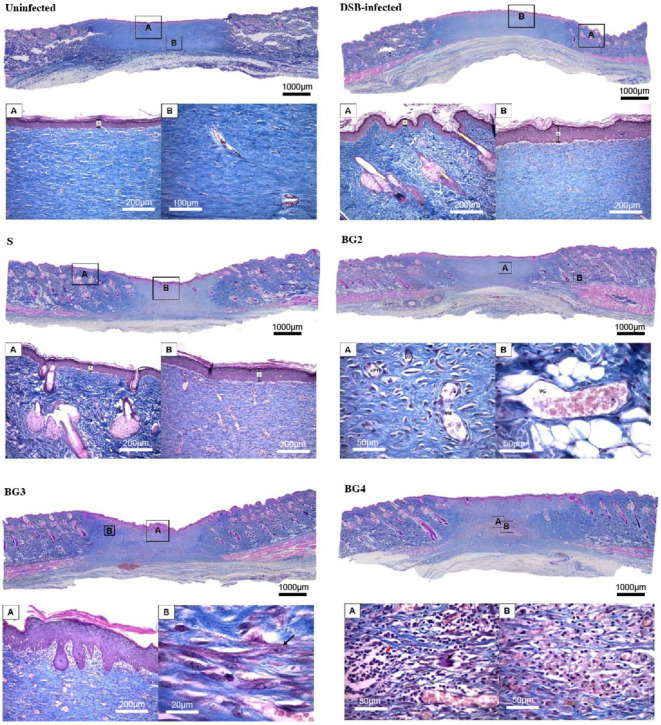
Representative histological images of wound tissue collected at the end of the treatment period (Day 12 post-treatment; Day 20 post-wounding) and stained with Arteta’s trichrome. For each condition, the upper panel shows a low-magnification overview (2.5×), while the lower images (**A**,**B**) show higher-magnification views of selected regions. Markers next to the epidermis indicate the region used for epidermal thickness measurement. In the BG3 sample, the black arrow indicates fibroblasts with their characteristic morphology (**B**). In the BG4 sample, abundant inflammatory infiltrate, predominantly lymphocytic, is observed (red arrow), along with a multinucleated giant cell (black arrow) (**A**). A diffuse macrophage infiltrate is also observed (*) (**B**). Scale bars are shown in each panel, and insets were acquired at different magnifications to highlight specific histological features.

**Figure 11:**
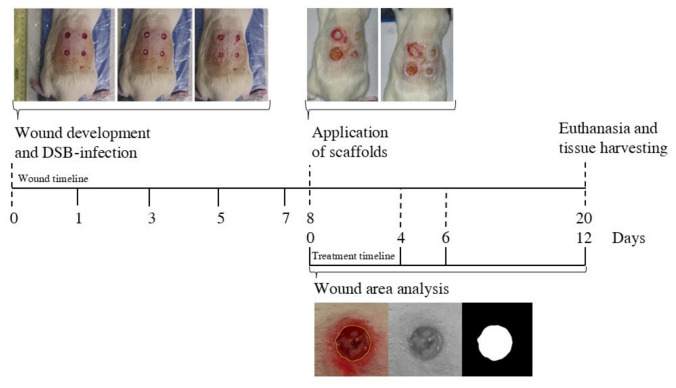
Timeline of in vivo evaluation of BG scaffolds in a murine model of DSB-infected wound.

Representative histological sections of the wound biopsies collected at the end of the treatment period are shown in Figure 10. Overall, the tissue architecture was characterized by a predominance of cellular components consistent with granulation tissue, with a variable deposition of the fibrillar matrix. In some samples, collagen fibers appeared sparse and loosely organized, whereas others show localized areas of increased fibrillar density. A density of blood vessels of varying diameters was observed, with a predominance of small-caliber vessels. An abundant inflammatory infiltrate was present, particularly in lesions that had not yet achieved closure. This infiltrate was mainly lymphocytic and distributed throughout the full thickness of the lesion, with edema and the presence of neutrophils observed in some cases. BG2 and BG3 displayed areas consistent with active repair and fibrillar matrix deposition, whereas BG4 showed a more cellular and inflammatory appearance, with abundant inflammatory infiltrate and less organized repair tissue. This observation is consistent with the poorer macroscopic healing response observed for BG4. Figure 11 summarizes the timing of wound inoculation, treatment, and tissue collection.

## 3. Discussion

### 3.1. Optimal Performance of BG2 and BG3 Scaffolds

The present study demonstrates that the incorporation of BG extract into polymeric scaffolds enhances their therapeutic performance in infected wounds, with BG2 and, to a lesser extent, BG3 showing the most favorable overall biological response. These formulations improved the wound closure kinetics (Figure 8), supported complete re-epithelialization (Figure 9), and exhibited more organized histological features (Figure 10) compared to both untreated wounds. Notably, BG2 showed the most consistent performance across endpoints, combining the significant wound area reduction at day 6 with complete re-epithelialization and an epidermal thickness comparable to uninfected tissue. Importantly, the control scaffold (S) also promoted favorable healing outcomes, consistent with its role as a moist and biocompatible wound scaffold [11,32]. In this context, the effect of BG-loaded scaffolds should be interpreted as an enhancement of an already beneficial scaffold environment rather than as an independent therapeutic effect. While BG3 improved wound closure at early time points compared with the control scaffold, it did not consistently outperform it in re-epithelialization, indicating that an increasing extract concentration does not linearly translate into improved biological outcomes.

The improved performance of BG2 likely reflects an optimal balance between antimicrobial activity and tissue compatibility. Although all BG-loaded scaffolds reduced bacterial viability in vitro (Figure 4), and BG3 showed the strongest reduction in dual-species biofilm viability (Figure 6), this did not result in proportional improvements in healing. This suggests that antimicrobial activity alone is insufficient to explain therapeutic performance and that host–material interactions play a central role in determining wound repair outcomes.

Histological and immunohistochemical findings support this interpretation (Figure 9 and Figure 10). BG2 and BG3 were associated with normalized epidermal architecture and controlled inflammatory responses, while maintaining adequate angiogenic and fibroblastic activity, as indicated by the VEGF and vimentin expression. Vimentin is important as it promotes fibroblast functions during the proliferative and remodeling phases of wound healing and also participates in pathogen clearance and extracellular matrix deposition [33] and it is known to have a reduced expression in chronic wounds as compared to acute wounds [34]. In contrast, BG4 showed an increased expression of these markers without corresponding improvements in healing, suggesting a dysregulated or prolonged repair process.

Further insight is provided by collagen deposition analysis (Figure 9E). BG4 exhibited the highest collagen-positive area, comparable to the control scaffold (S). This effect may be related to the biological activity of the BG extract, which has been shown to stimulate fibroblast growth in vitro, a process closely associated with dermal matrix formation [19,35]. However, this increased collagen deposition did not translate into improved wound closure (Figure 8) or re-epithelialization (Figure 9A), which suggests that collagen deposition alone does not necessarily indicate effective tissue repair and may reflect dysregulated matrix remodeling in the context of persistent inflammation.

Consistent with this interpretation, BG4-treated wounds displayed a more pronounced inflammatory pattern, characterized by abundant cellular infiltration and a less organized tissue structure (Figure 10). Although BG extracts and their polyphenolic components are generally associated with anti-inflammatory and antioxidant effects [18], there is no evidence supporting direct pro-inflammatory activity. Therefore, the observed response in BG4 is more likely related to an unfavorable local microenvironment generated by excessive extract loading, rather than an intrinsic inflammatory effect of the extract itself. In contrast, intermediate extract concentrations (BG2 and BG3) were associated with a more controlled inflammatory response, supporting a concentration-dependent biological effect.

A limitation of the present study is that multiple wounds were generated within the same animal, which may introduce intra-animal correlation between observations. However, this design also allowed the randomization of treatments within each animal while reducing the total number of animals required, consistent with the principles of reduction in animal experimentation [36]. Therefore, although the results should be interpreted cautiously regarding the strict statistical independence between wounds, the model enabled a comparative evaluation of multiple scaffold formulations under shared systemic physiological conditions.

### 3.2. Impact of Dual-Species Biofilm Infection on Wound Healing

The establishment of a DSB infection model using *P. aeruginosa* and *S. aureus* successfully reproduced key features of chronic wound infection, including delayed healing, persistent inflammation, and impaired tissue regeneration (Figure 6). Infected wounds exhibited an approximately 80% reduction in wound closure compared to uninfected controls, confirming the significant impact of biofilm-associated infections on healing progression. This impairment is consistent with the known pathogenic synergy between these microorganisms. *P. aeruginosa* can form deeply embedded biofilms within the wound bed, while *S. aureus* is often localized in more superficial layers [23,24]. These results also reproduced what was reported by Dalton et al. [37], who established an in vivo model of infection from an in vitro biofilm formation. Their four-species biofilm resulted in a higher impairment in wound healing compared to monospecies biofilms.

Their coexistence enhances the biofilm robustness, increases the tolerance to antimicrobial agents, and sustains a pro-inflammatory microenvironment [28,29]. In the present study, this was reflected not only by macroscopic signs of infection, including redness and the presence of yellowish exudate observed in several wounds (Figure 8A), but also at the tissue level by the presence of abundant inflammatory infiltrates, increased epidermal hyperplasia, and altered expression of key markers such as pan-cytokeratin and vWF in infected wounds (Figure 9 and Figure 10).

Interestingly, bacterial counts did not differ significantly between infected and uninfected wounds at the time of analysis (Figure 7C). Similarly, Dalton et al., also found that the bacterial load remained constant despite the impact on wound closure [37]. The observed differences in healing outcomes highlight that biofilm-mediated pathogenicity is not solely dependent on the bacterial load but also on the bacterial organization and host response modulation. An important limitation of the present study is that the bacterial burden was quantified only at the end of the treatment period. Because the infected wound model was established in otherwise healthy immunocompetent animals, substantial host-driven bacterial clearance may have occurred. Therefore, the endpoint CFU counts may not fully reflect the bacterial burden during the active phase of infection. Nevertheless, the infected wounds still showed delayed closure, persistent inflammatory features, and altered tissue organization compared with uninfected wounds, indicating that the dual-species biofilm model produced a biologically relevant impairment of healing. Therefore, the present findings suggest that the therapeutic effect of BG-loaded scaffolds likely results from a combination of scaffold-mediated wound support and concentration-dependent biological activity of the extract, rather than from antimicrobial activity alone. Earlier sampling time points, as well as the use of impaired-healing models such as diabetic animals [38], would likely provide a more accurate evaluation of the antimicrobial contribution of the BG extract during the active infection phase.

The persistence of inflammatory cells and the delayed re-epithelialization observed in infected wounds further support the role of biofilms in maintaining wounds in a chronic inflammatory state [39,40]. Overall, our DSB model provides a relevant and simpler platform for evaluating antimicrobial and regenerative therapies, as it captures the complexity of polymicrobial infections commonly observed in chronic wounds.

### 3.3. Influence of Physicochemical Properties and Extract–Polymer Interactions

The incorporation of the BG extract significantly influenced the physicochemical properties of the scaffolds, which, in turn, impacted their biological performance. Increasing the extract concentration was associated with a reduced pore size, moisture content, and swelling capacity, as well as decreased mechanical resistance, particularly in the BG4 formulation (Figure 3). These changes are likely related to the interactions between BG extract components, such as polyphenols, and scaffold polymers, particularly gelatin. Polyphenols, such as quercetin and verbascoside, are known to interact with proteins through hydrogen bonding and hydrophobic interactions, which can modify protein conformation and promote matrix compaction [41,42]. In our previous work [31], Raman spectroscopy analysis provided evidence of interactions between quercetin and gelatin, suggesting the formation of non-covalent complexes within the scaffold network. While moderate interactions (BG2 and BG3) may enhance scaffold stability and control fluid uptake, excessive interactions, as seen in BG4, may compromise the structural integrity, resulting in friability, a reduced pore size, and reduced mechanical performance.

The structural changes associated with higher extract loading have important implications for tissue repair. The pore size and porosity are critical parameters for cell infiltration and tissue regeneration. Larger and interconnected pores facilitate fibroblast migration, vascularization, and nutrient diffusion, whereas smaller pores may limit cell penetration and reduce scaffold biocompatibility. Choi et al. found significant higher proliferation with pores of about 580 µm versus 435 µm [43]. Our formulations had significantly smaller pores with values of around 100 to 250 µm for the BG2 to BG3 scaffolds, which had better in vivo performance compared to BG4 with an average pore size of below 30 µm (Figure 2 and Figure 3). In this study, BG4 exhibited the smallest pore size and poorest mechanical properties, which may have contributed to its inferior in vivo performance despite the strong antimicrobial activity. Although the fibroblast viability remained above 80% across all formulations, suggesting no overt cytotoxicity (Figure 5), the reduced porosity and structural integrity of BG4 may have limited effective cell–scaffold interactions.

Additionally, the reduced swelling capacity observed with an increasing extract content may influence the ability of scaffolds to maintain a moist wound environment, which is essential for optimal healing. Scaffolds with moderate swelling (BG2 and BG3) likely provided a better balance between fluid retention and structural stability, supporting both cell activity and wound hydration. In addition, only the control scaffold (S) and BG1 showed visible degradation after 24 h in the in vitro bacterial incubation assay, whereas scaffolds with higher BG loading appeared more structurally preserved (Supplementary Figure S2). Although qualitative, this observation suggests that BG incorporation may influence the matrix stability and degradation behavior.

Preliminary release experiments performed with BG1 and BG3 confirmed the measurable release of flavonoid-containing compounds from the scaffolds in both PBS and bovine plasma during the first 6 h (Supplementary Figure S1). The release tended to be higher in bovine plasma than PBS and greater for BG3 than BG1, indicating that both scaffold loading and the surrounding biological environment influence the extract availability. However, because BG4 was not included in this assay, these findings only provide indirect support for the scaffold-mediated release behavior.

Taken together, these findings highlight that the therapeutic performance of BG-loaded scaffolds is governed not only by the biological activity of the extract but also by its impact on the physicochemical properties of the delivery system. Achieving an optimal balance between the antimicrobial efficacy, mechanical integrity, and structural properties is essential for maximizing wound healing outcomes. Further studies including comprehensive release kinetics, degradation profiling, and the direct evaluation of cell infiltration are required in order to fully elucidate the mechanisms underlying these observations.

## 4. Materials and Methods

### 4.1. Materials

Gelatin from bovine skin type B (MW range 50–100 kDa), resazurin, fluorescein diacetate (FDA), N-hydroxysuccinimide (NHS), N-(3-dimethylaminopropyl)-N′-ethylcarbodiimide hydrochloride (EDC HCl), MES hydrate, quercetin, phosphate-buffered saline (PBS), bovine plasma, and diaminobenzidine (DAB) were obtained from Sigma Aldrich (St. Louis, MO, USA). Chitosan from crab shells (MW range of 250–350 kDa and a 98% deacetylation degree) was provided from Quitoquimica (Coronel, Chile). Sodium hyaluronate (MW range 100 to 1800 kDa) was obtained from Lifecore Biomedical (Chaska, MN, USA).

### 4.2. Scaffold Development

Previously, we studied the effect of the polymeric composition of scaffolds consisting of chitosan, gelatin, and hyaluronic acid on their physicochemical and biological properties and compared these changes with respect to a reference scaffold [32]. We identified a polymeric composition that optimized these properties [31], and we used that scaffold formulation as a base to develop 4 new scaffolds containing different concentrations of a standardized hydroalcoholic *Buddleja globosa* Hope (BG) extract (kindly donated by Laboratorios Ximena Polanco, Santiago, Chile). This BG extract has a total phenol content of 7378 µg/mL, expressed as catechin, flavonoid content of 24,024 µg/mL expressed as quercetin, and 3696 µg/mL of verbascoside [31,44].

Briefly, stock solutions of chitosan, hyaluronic acid, and gelatin were prepared in purified water and then were combined in 9 cm Petri dishes according to the optimized proportion established by Ceriani et al. [31], plus four different BG extract concentrations (BG1, BG2, BG3, and BG4). Additionally, a reference scaffold without BG extract (named S) was also developed, representing the reference scaffold according to the reports of Enrione et al. [32]. Table 2 shows the scaffold compositions, indicating the volumes of the polymer stock solutions used for each scaffold and the BG content expressed as verbascoside concentration.

The Petri dishes were gradually frozen by storing them for sequential 24 h in refrigeration (2–8 °C), freezing (−20 °C), and, finally, at ultra-freezing (−70 °C) until lyophilization. Then, the scaffolds were crosslinked as previously described [31]. After a second lyophilization, scaffolds were UV-sterilized and stored protected from the light at room temperature until further evaluation.

### 4.3. Physical Properties of the BG Scaffolds

Pore areas were characterized by scanning electron microscopy (SEM). Cross-sectional images of each scaffold were acquired using a field-emission SEM (Quattro S, ThermoFisher Scientific Inc., Waltham, MA, USA) operating in environmental mode (ESEM) with a gaseous secondary electron detector (GSED). Image analysis was performed with Fiji ImageJ (version 1.54f) [45] employing the Trainable Weka Segmentation (TWS) plugin. This machine-learning-based approach allowed consistent identification and segmentation of pore boundaries through an iterative process that included manual annotation during training. The measured pore areas were subsequently converted into equivalent circular diameters for standardized comparison.

For moisture determination, samples of scaffolds (≥0.5 g) were tested in a halogen moisture analyzer (HE53, Mettler Toledo, Leicester, UK) at 160 °C for 10 min. The initial weight of the sample (W_initial_) and the weight at the end of the drying process (W_final_) were used to determine the % moisture content of the scaffolds as follows:

(1) $$Moisture\;content(\%)=\frac{W_{initial}-W_{final}}{W_{initial}}\times 100,$$

For swelling behavior determination, scaffold pieces of a diameter of 8 mm were weighed (W_dry_) and placed in tubes with 1 mL PBS at room temperature for 24 h. Then, the excess PBS was gently removed by placing them in sterile filter paper and then weighed (W_wet_). The %swelling was calculated as follows:

(2) $$Swelling\;ratio(\%)=\frac{W_{wet}-W_{dry}}{W_{dry}}\times 100,$$

Scaffold strips of 10 × 40 mm were studied in a semiautomatic laboratory-made folding endurance tester. The tester consists of two clamps that hold a piece of material in a vertical position, with a bottom 180° oscillatory motorized clamp and an upper fixed clamp that lifts the material with an adjustable counterweight. The instruments report the number of folds until the material breaks and is lifted by the upper clamp, which stops the counter. A total of 1000 folds were set as the maximum folds, and each scaffold was tested in triplicates.

Finally, preliminary release experiments were performed using BG1 and BG3 scaffolds as representative low- and intermediate-/high-loading formulations, respectively. Scaffolds pieces (1 × 1 cm) were incubated in 3 mL PBS or bovine plasma at 37 °C and aliquots were collected after 0.5, 1, 3, and 6 h of incubation. Total flavonoid content released into the surrounding media was estimated using a total flavonoid assay and expressed as quercetin equivalents (QEs). Briefly, collected samples were filtered and incubated with aluminum chloride; then, ethanolic sodium hydroxide solution was added. The resulting absorbance was measured at 510 nm using UV–Vis spectrophotometry. Flavonoid concentration was calculated from a quercetin calibration curve (R^2^ = 0.9962; from 35.8 to 358 µg/mL) and expressed as quercetin equivalents (QE). Control scaffolds without BG extract incubated under the same conditions were used as analytical blanks.

### 4.4. Antimicrobial Properties and Cytocompatibility In Vitro

*Pseudomonas aeruginosa* (ATCC 27853) and *Staphylococcus aureus* (ATCC 29213) were stored in frozen stocks in LB or TSB broth, respectively, with 50% *v*/*v* glycerol. For each experiment, suspension cultures were prepared with 3 to 5 colonies from overnight agar cultures in LB or TSA agar at 37 °C. The suspension was incubated for 24 h before adjusting the concentration to 1–3 × 10^6^ CFU/mL.

BG scaffolds (8 mm diameter) were submerged and incubated in 200 µL of the adjusted bacterial suspension described above at 37 °C and 75 rpm for 24 h. After incubation, the remaining scaffolds were rinsed twice with PBS and placed in a black 96-well plate, while the plate used for the treatment was stained with crystal violet for bacterial adhesion determination. For bacterial viability within the scaffolds, each well was filled with 200 μL of fluorescein diacetate (FDA) for *P. aeruginosa* or resazurin for *S. aureus* [46]. The plate was protected from light and incubated at 37 °C and 30 min. FDA fluorescence was recorded at 494 nm (excitation) and 518 nm (emission) and resazurin fluorescence at 560 nm (excitation) and 518 nm (emission). Finally, the wells were rinsed twice with PBS and then fixed with methanol. Then, 0.1% *w*/*v* crystal violet was used for 30 min and rinsed with water before air-drying overnight. Crystal violet retained in the wells was dissolved in 30% *v*/*v* acetic acid, and the absorbance was read at 540 nm.

Human dermal fibroblasts (Life Technologies Corp., Grand Island, NY, USA) were cultured in Dulbecco’s Modified Eagle Medium (DMEM) supplemented with 10% fetal bovine serum (FBS; Cytiva, Marlborough, MA, USA), 100 U/mL penicillin, 10 µg/mL streptomycin, and 2 mM Glutamax (Thermo Fisher Scientific, Waltham, MA, USA). Cells (passages 5 to 7) were maintained under standard culture conditions in a humidified incubator at 37 °C with 5% CO_2_. BG scaffolds (8 mm diameter) were placed in a microtiter plate, and the cells were then seeded onto each scaffold at a density of 1 × 10^4^ cells per sample.

Cell viability was evaluated after 3 days using a resazurin reduction assay [47]. Samples were incubated with a 4 mg/mL resazurin solution for 4 h, after which fluorescence was recorded at excitation/emission wavelengths of 560/590 nm using a microplate reader (Varioskan, Thermo Fisher Scientific, Waltham, MA, USA).

### 4.5. Scaffold Efficacy in In Vitro Infected Wound Model

*P. aeruginosa* and *S. aureus* biofilms were developed according to the model reported by Sun et al. [48] with modifications. Overnight suspension cultures of both species were harvested by centrifugation, resuspended in Bolton broth, and adjusted to 1–3 × 10^6^ CFU/mL. Then, the adjusted suspensions were combined in equal volumes to form the mixed-species inoculum. A tube containing 6 mL of supplemented Bolton broth (50% *v*/*v* bovine plasma, 1% *w*/*v* agar, and 2% *w*/*v* gelatin) was inoculated with 10 µL of the mixed suspension by ejecting the tip of the pipette, which served as a growth surface. After 24 h of incubation at 75 rpm and 37 °C, the mixed biofilm was harvested and rinsed in PBS. The biofilms had average masses of 0.49 ± 0.21 g. This biofilm model was used to inoculate the artificial wound beds and the animals.

Artificial wound beds were developed according to Kucera et al. [49]. First, 3 sterile stainless-steel rods (2 × 8 mm) were placed over a layer of agar (1.2%) and gelatin (1%) in 9 mm Petri dishes. Then, another layer of agar and gelatin was poured over until it almost covered the rods. The rods were removed, and pre-weighed biofilm pieces (0.03 ± 0.01 g) were placed in each of the wounds made by the rods. After 24 h of incubation, the biofilms were removed, and scaffold pieces (10 × 20 mm) moistened in sterile saline solution were placed over the wound before incubation at 37 °C for 24 h. Finally, pieces of the artificial wound bed with the remaining scaffolds were collected using a scalpel and homogenized in 10 mL PBS for microbial enumeration by the drop-plate method [50].

### 4.6. Scaffold Efficacy in an In Vivo Infected Wound Model

All experiments and procedures with animals, including surgery, wound cleaning, anesthesia, and euthanasia, were carried out by veterinary doctors following the protocols SBEA148 which was approved in 2019 and revised in 2024 by the Institutional Animal Care and Use Committee (IACUC) of the University of Valparaíso.

Sprague Dawley male rats (average weight of 350 g) were acclimated for a week before the experiments. Before the evaluation of the efficacy of the scaffolds, a group of animals (n = 6) were selected for establishment of the DSB-infection model. Then, a group of DSB-infected animals (n = 11) was dedicated to evaluating the efficacy of the scaffolds in this wound infection model.

All animals received four 8 mm full-thickness wounds in the palpebral skin using a biopsy punch under anesthesia with isoflurane. These wounds (n = 44) were inoculated with about 30 mg (±15%) of DSB following the procedure of Dalton et al. [37] but with modifications and maintained in position for 7 days with a transparent wound film (Tegarm Film^®^, 3M, Brookings, SD, USA) (Figure 11). On day 8, wounds were cleaned and received one of the following randomized treatments: (1) untreated, (2) S (without BG extract), (3) BG2, (4) BG3, and (5) BG4. Scaffolds were applied in pieces of 8 mm after being moistened in sterile saline solution, placed in the cleaned wounds, and held with a transparent wound film. Then, animals were monitored daily with photography on days 4, 6, and 12. Then, the animals were euthanized and wound biopsies were collected and stored in PBS for microbial enumeration or fixed with 10% paraformaldehyde pH 7.0 for histology analysis and microscopy.

### 4.7. Wound Closure Analysis

Animals were photographed every 1 or 2 days using a standard height next to a ruler for image analysis using Fiji ImageJ (NIH, Bethesda, MD, USA) employing the Trainable Weka Segmentation (TWS) plugin for wound area detection and measurement. The wound area on day 8 of infection (right before application of the scaffolds) represented the initial area (100%).

### 4.8. Microbial Enumeration and Histological Analysis

For microbial enumeration, wound biopsies were weighed and homogenized in 10 mL of PBS before serial dilution and drop-plating in selective agars, using *Pseudomonas* CN agar for *P. aeruginosa* and Baird–Parker agar for *S. aureus*.

### 4.9. Histological Analysis

Preserved wound biopsies were processed using a Leica TP1020 tissue processor (Nussloch, Germany), undergoing infiltration with Paraplast Plus. The samples were embedded in Paraplast Plus at 60 °C using a Leica EG1150H embedding center and solidified on a Leica EG1150C cooling plate. Using a Leica RM2155 microtome, ribbons of transverse sections (5 µm thickness) were obtained. Selected sections (two per lesion) were stretched in a flotation bath with distilled water at 40 °C and mounted on poly-l-lysine-coated slides. Slides were then dried in an oven at 37 °C for 24 h.

Arteta’s trichrome-staining method [51] was used as a topographic stain to identify the morphology of the epidermis, dermis, and their cellular and fibrillar components. All samples were examined under a microscope at 2.5× magnification for overview, 10× to determine the number of epidermal cell layers, collagen deposition, and the thickness of hyperplastic and normal epidermis, and 40× for determination of the number of inflammatory cells using ImageJ. Collagen deposition was also quantified from these sections. For each sample, 3 to 5 non-overlapping fields within the dermal wound region were selected, avoiding areas with staining artifacts, folds, tears, or tissue detachment. Images were analyzed using the Color Deconvolution plugin to separate the collagen-associated staining signal, followed by thresholding to identify collagen-positive regions. Collagen deposition was expressed as the percentage of collagen-positive area relative to the total tissue area analyzed [52].

Immunohistochemical staining was performed for each experimental condition. Antigen retrieval was carried out by heat using a steamer. Endogenous peroxidase activity was blocked with 2% H_2_O_2_ in methanol, followed by blocking with 2% horse serum in Tris buffer. Samples were incubated overnight at 8 °C in a humid chamber with primary antibodies. The following day, sections were incubated with a biotinylated anti-mouse secondary antibody produced in horse (BA-2000, Vector Laboratories, Newark, CA, USA; dilution 1:500). The standard ABC peroxidase kit (Vector Laboratories) was used, and DAB served as the chromogen. The following antigens were evaluated: (1) Pan-cytokeratins (AE1/AE3, Santa Cruz, Dallas, TX, USA, mouse monoclonal, 1:500 dilution), antigen retrieval: Tris-EDTA buffer, pH 9, 15 min (steamer), (2) Vimentin (V9, Santa Cruz, mouse monoclonal, 1:1000 dilution), Antigen retrieval: Tris-EDTA buffer, pH 9, 15 min (steamer), (3) Vascular Endothelial Growth Factor (VEGF) (C1, Santa Cruz, mouse monoclonal, 1:300 dilution), Antigen retrieval: Tris-EDTA buffer, pH 9, 15 min (steamer), and (4) Von Willebrand Factor (vWF) (F8/86, Santa Cruz, mouse monoclonal, 1:300 dilution), Antigen retrieval: Citrate buffer, pH 6, 15 min (steamer).

### 4.10. Statistical Analysis

Data were statistically analyzed using JMP^®^ software (Version 19, Student Edition, SAS Institute Inc., Cary, NC, USA). One-way analysis of variance (ANOVA) was performed, followed by Tukey’s post hoc test for multiple comparisons and Student’s *t*-test for pairwise comparisons with a significance level set at α = 0.05.

## 5. Conclusions

This study demonstrates that polymeric scaffolds incorporating *Buddleja globosa* extract represent a promising strategy for the treatment of biofilm-infected wounds. Among the formulations evaluated, BG2 achieved the most consistent overall performance, combining improved wound closure, complete re-epithelialization, and favorable physicochemical properties. BG3 also improved early wound closure but did not consistently outperform the control scaffold in histological endpoints.

The DSB model successfully reproduced key features of chronic wound infection, including delayed healing and sustained inflammation, highlighting the importance of targeting biofilm-associated pathogens in wound care strategies. While an increasing extract content enhanced antimicrobial activity, excessive incorporation, as observed in BG4, negatively affected the scaffold structure and performance, underscoring the need for the careful optimization of the formulation parameters.

Overall, the findings indicate that the therapeutic efficacy is not only determined by the antimicrobial potency but depends on achieving an appropriate balance between extract loading and scaffold functionality. Further studies are required to better characterize the extract release kinetics and to define the optimal dosing and reapplication strategies for clinical translation.

## Figures and Tables

**Table 1:** Scaffold properties.

Scaffold	Mass (mg)	Density (g/mL)	BG Content Expressed as Verbascoside (mg/g)
S	241.1 ± 1.6	0.017 ± 0.001	0
BG1	305.2 ± 1.2	0.024 ± 0.001	0.06
BG2	305.3 ± 2.7	0.024 ± 0.001	0.7
BG3	319.0 ± 0.7	0.026 ± 0.001	6.4
BG4	517.3 ± 7.6	0.040 ± 0.001	50

S: reference scaffold; and BG1 to BG4: scaffolds containing increasing concentration of BG extract.

**Table 2:** Scaffolds polymeric and BG extract composition.

Scaffold	C (mL)	HA (mL)	G (mL)	BG Content Expressed as Verbascoside (µg/mL)
S	3.8	1.9	13.3	0
BG1	5.7	0.95	13.3	1
BG2	5.7	0.95	13.3	10
BG3	5.7	0.95	13.3	100
BG4	5.7	0.95	13.3	1000

S: reference scaffold; BG1 to BG4: scaffolds containing increasing concentration of BG extract; C: chitosan; HA: hyaluronic acid; and G: gelatin.

## Data Availability

The dataset will be available upon request from the authors.

## Supplementary Materials

The following supporting information can be downloaded at: https://www.mdpi.com/article/10.3390/ijms27104240/s1.

## Abbreviations

The following abbreviations are used in this manuscript:

BG	* Buddleja globosa *
BG1 to BG4	Scaffolds containing increasing concentrations of BG extract
CFU	Colony-forming units
DAB	Diaminobenzidine
DMEM	Dulbecco’s Modified Eagle Medium
DSB	Dual-species biofilm
EDC HCl	N-(3-dimethylaminopropyl)-N′-ethylcarbodiimide hydrochloride
FDA	Fluorescein diacetate
FESEM	Field-Emission Scanning Electron Microscopy
LB	Luria–Bertani
MES	2-(N-morpholino) ethanesulfonic acid
NHS	N-hydroxysuccinimide
PBS	Phosphate saline buffer
S	Reference scaffold
TSA	Tryptic soy agar
TWS	Trainable Weka Segmentation
VEGF	Vascular endothelial growth factor
vWF	Von Willebrand Factor
