# Supplementary figures and images for: Optimization of Buddleja globosa-Loaded Polymeric Scaffolds for the Treatment of Biofilm-Infected Wounds
Source: Int J Mol Sci. 2026 May 10;27(10):4240. doi: 10.3390/ijms27104240 (PMC13207814; doi:10.3390/ijms27104240)

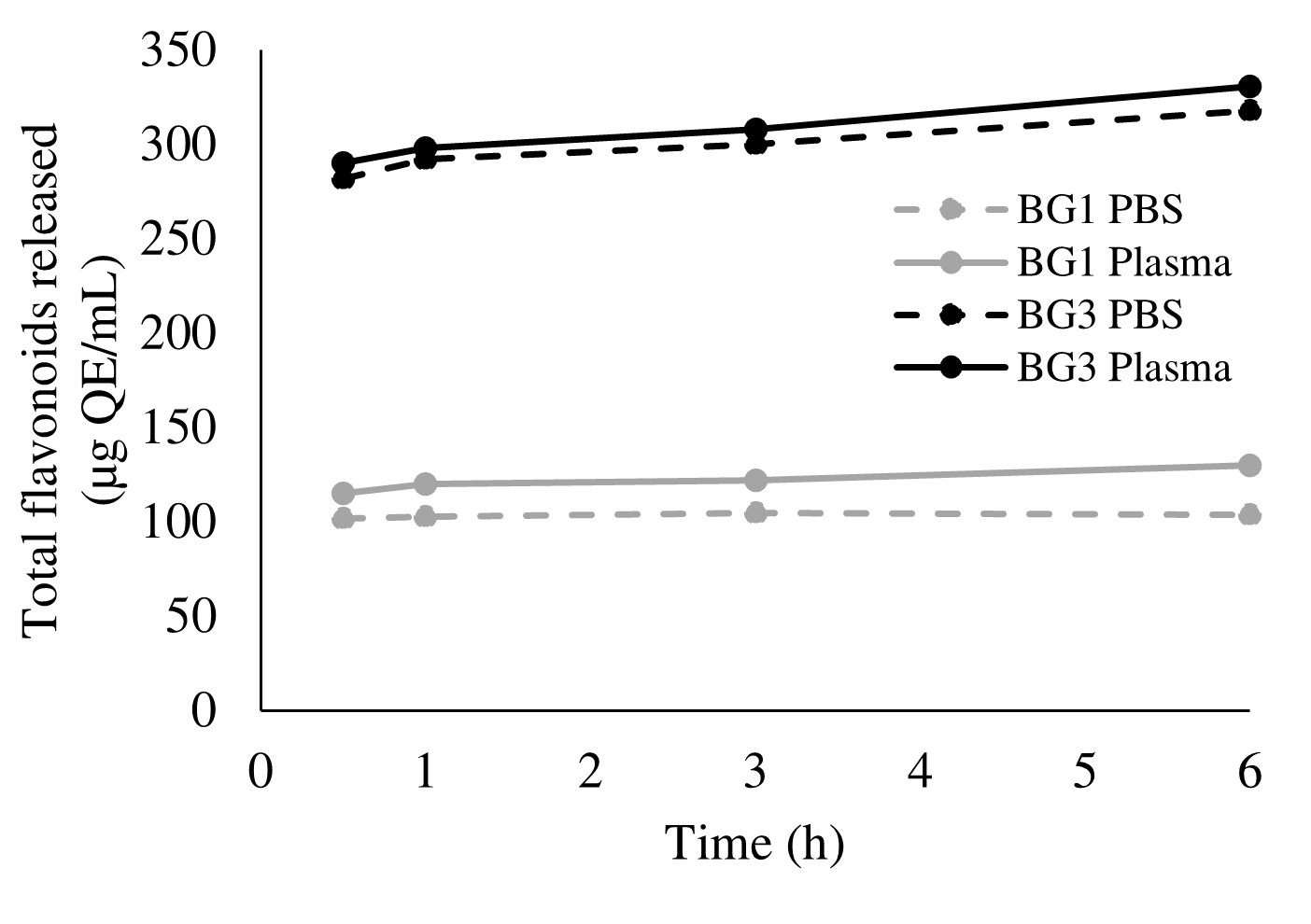

Supplement: Supplementary file 1 [file ijms-27-04240-s001.zip › Sup Figure S1. Release.jpg]

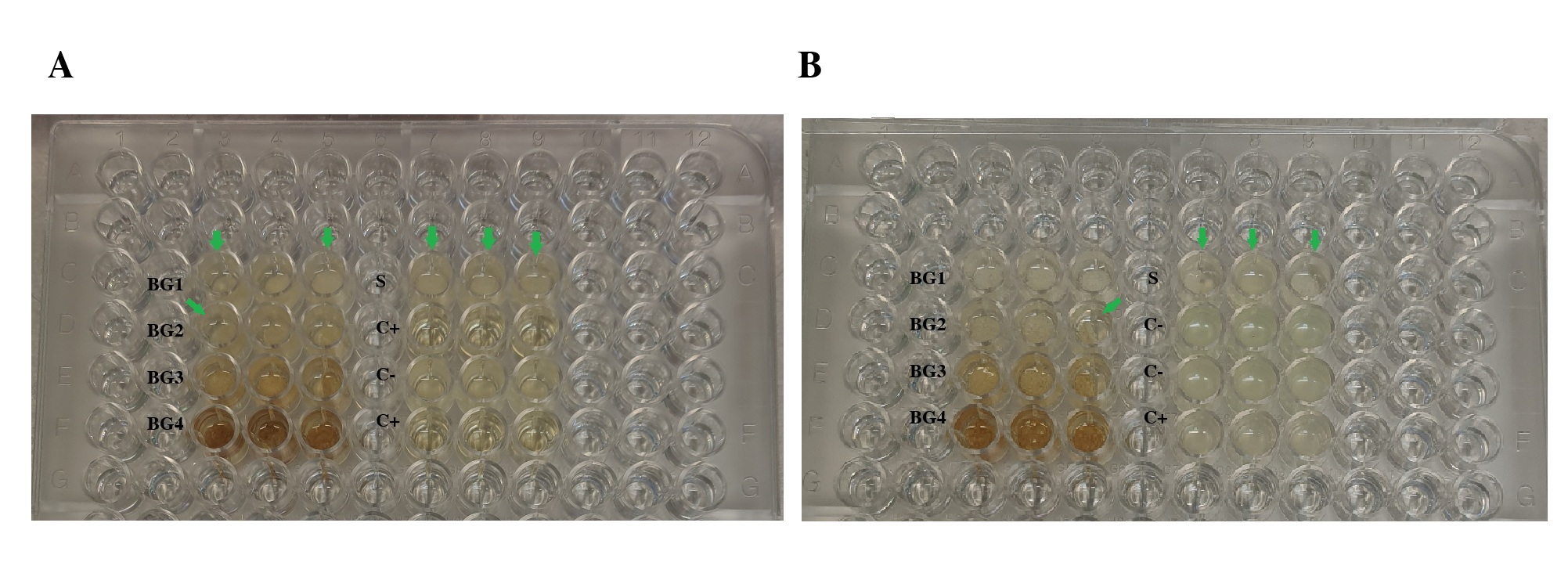

Supplement: Supplementary file 1 [file ijms-27-04240-s001.zip › Sup Figure S2. Scaffolds appearance after treatment.jpg]

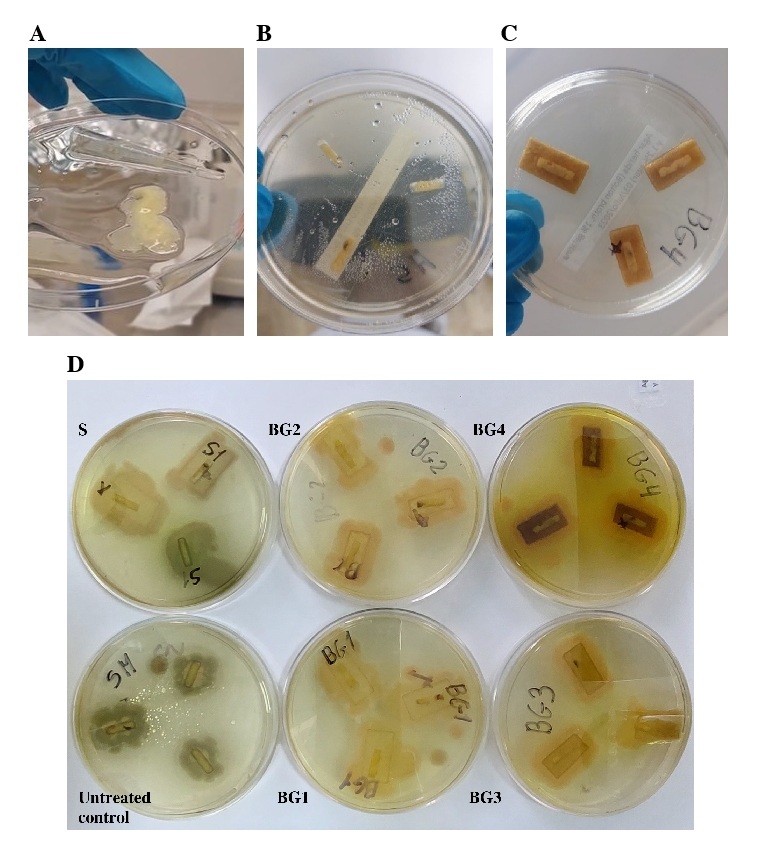

Supplement: Supplementary file 1 [file ijms-27-04240-s001.zip › Sup Figure S3. In vitro DSB treatment.jpg]

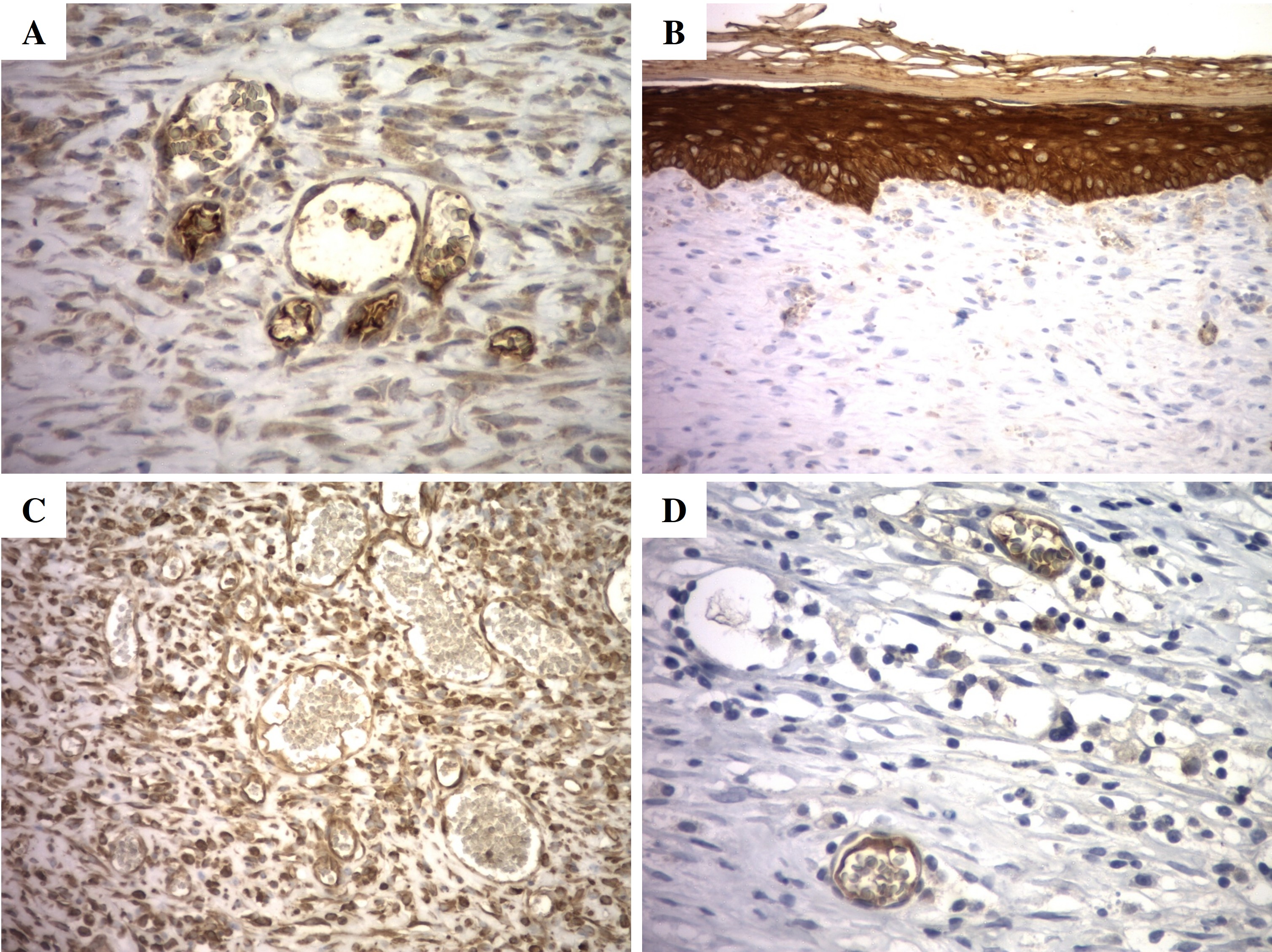

Supplement: Supplementary file 1 [file ijms-27-04240-s001.zip › Sup Figure S4. Immunestaining.jpg]
